# Distinct Gut Microbiome Signatures in Hemodialysis and Kidney Transplant Populations

**DOI:** 10.3390/jcm14228032

**Published:** 2025-11-12

**Authors:** Luminita Voroneanu, Andreea Covic, Stefan Iliescu, Cezar Valeriu Baluta, Bogdan Dumitru Agavriloaei, Anca Elena Stefan, Roxana-Maria Amărandi, Irina-Cezara Văcărean-Trandafir, Iuliu-Cristian Ivanov, Adrian Covic

**Affiliations:** 1 Faculty of Medicine, University of Medicine and Pharmacy “Grigore T Popa”, 700115 Iasi, Romania; lumivoro@yahoo.com (L.V.); stefiliescu@yahoo.fr (S.I.); baluta_cezar@yahoo.com (C.V.B.); bogdan.agavriloaei@yahoo.com (B.D.A.); accovic@gmail.com (A.C.); 2 Nephrology Clinic, Dialysis, and Renal Transplant Center, “C.I. Parhon” University Hospital, 700503 Iasi, Romania; 3 Faculty of Automatics, Technical University of Sofia, Kliment Ohridski Boulevard 8, 1756 Sofia, Bulgaria; roxana-maria.amarandi@tu-sofia.bg; 4 TRANSCEND Research Centre, Regional Institute of Oncology, 2-4 General Henri Mathias Berthelot Street, 700483 Iasi, Romania; irina.trandafir@iroiasi.ro (I.-C.V.-T.); iuliu.ivanov@iroiasi.ro (I.-C.I.)

**Keywords:** gut microbiota, chronic kidney disease, hemodialysis, kidney transplantation, dysbiosis

## Abstract

**Highlights:**

- Gut microbiota composition differs markedly between hemodialysis (HD) patients, kidney transplant recipients (KTR), and healthy controls (HC).
- HD patients showed enrichment of *Akkermansia* and *Clostridia*-related taxa, while KTR exhibited partial recovery of SCFA-producing genera such as *Faecalibacterium* and *Roseburia*.
- Microbial diversity was higher in HD compared with KTR, but both groups displayed dysbiosis relative to healthy controls.
- Clinical status, rather than diet or serum biomarkers, was the primary determinant of microbiome variation.

**Abstract:**

**Background:** Gut microbiota plays a critical role in host metabolism, immunity, and intestinal barrier integrity. Both chronic kidney disease (CKD) and kidney transplantation (KTR) are associated with gut dysbiosis, driven by uremic toxins, comorbidities, and immunosuppressive therapy. However, direct comparisons between hemodialysis (HD), KTR, and healthy controls (HC), while accounting for dietary factors, remain limited. **Methods:** We conducted a cross-sectional study including 48 HD patients, 75 KTR patients, and 13 HC. Stool patient samples were analyzed using 16S rRNA amplicon sequencing targeting the V4-V4 region to assess microbial composition and diversity. Data on clinical status, laboratory parameters, and dietary intake were collected and integrated with microbiome profiling. **Results:** Firmicutes and Bacteroidota dominated all groups, with *Akkermansia* enriched in HD and SCFA-producing genera (*Faecalibacterium, Roseburia*) more abundant in KTR. LEfSe and sPLS-DA analyses identified *Akkermansia* and *Clostridia*-related taxa as discriminants of HD, while *Acidaminococcus* and *Megasphaera* characterized KTR. HD patients exhibited higher alpha diversity (Faith’s PD and Chao1) than KTR (*p* < 0.05). Dietary intake differed across groups, but explained only a small proportion of microbial variance. **Conclusions:** Both HD and KTR patients display persistent gut dysbiosis with distinct microbial signatures. While transplantation partially restores SCFA producers, immunosuppression and diet shape new ecological shifts. These findings underscore the potential of microbiota as a biomarker and therapeutic target in renal replacement therapies.

## 1. Introduction

The understanding of gut microbiota has significantly expanded, particularly in the areas of metabolism, inflammation, and immunology. The human gut ecosystem comprises trillions of microorganisms, evolving into a veritable metabolically active organ [1]. The most dominant bacterial phyla in the gut are Firmicutes and Bacteroidetes, Actinobacteria, and Proteobacteria, along with Cyanobacteria and Spirochaetes [2]. This balanced microbial community plays essential roles in nutrient metabolism, immune modulation, maintenance of gut barrier integrity, and overall metabolic health by producing short-chain fatty acids (SCFAs) and synthesizing vitamins [3]. In chronic kidney disease (CKD), gut dysbiosis arises from factors such as multidrug therapy, comorbidities, uremic milieu, low-fiber diets, constipation, and ageing [4]. Impaired kidney function leads to the accumulation of uremic toxins (UT) due to decreased excretion, increased production, or both. Uremic toxins (UT) are biologically active compounds that accumulate as kidney function declines. They primarily originate from gut microbial metabolism of dietary amino acids such as tryptophan, tyrosine, and choline, generating precursors such as indole and p-cresol, which are subsequently converted in the liver into indoxyl sulfate (IS) and p-cresyl sulfate (pCS). According to the European Uremic Toxin Work Group (EUTox) classification, UT are grouped into small water-soluble molecules, protein-bound compounds, and middle molecules, with protein-bound uremic toxins (PBUTs) being particularly difficult to remove by dialysis [5]. Experimental and human data indicate that elevated plasma UT levels alter the gut microenvironment by increasing luminal pH, damaging epithelial tight junctions, and promoting the overgrowth of UT-producing bacterial taxa, thus amplifying gut dysbiosis [6,7]. This relationship is bidirectional: uremia disrupts the intestinal barrier and microbial balance, while dysbiosis further increases intestinal generation and systemic absorption of UT. Recent studies have demonstrated that uremia impairs epithelial integrity and tight junctions, facilitating the translocation of endotoxins and microbial fragments into the circulation, fueling systemic inflammation, oxidative stress, and further renal and cardiovascular damage [8,9,10]. CKD-associated dysbiosis consistently exhibits reduced diversity, characterized by the depletion of beneficial genera (e.g., *Roseburia*, *Bacteroides*, *Prevotella*) and the enrichment of pathogenic taxa (*Klebsiella*, *Enterobacteriaceae*, *Fusobacterium*). This dysbiosis contributes to impaired gut health, systemic inflammation, and further renal and cardiovascular damage [11].

In kidney transplantation, dysbiosis is driven by surgical stress, antibiotics, immunosuppression, and dietary changes, leading to reduced diversity, loss of baseline dominant taxa, and emergence of new species, which have been linked to diarrhea and infections [12]. Immunosuppressive therapy, commonly used in KT, has a profound and multifaceted impact on the gut microbiota and intestinal barrier. These agents have been shown to significantly alter the intestinal microenvironment by affecting mucus secretion, epithelial permeability, mucosal immunity, and decreasing microbial diversity [13,14].

Given the increasing evidence supporting the impact of gut microbiota on clinical outcomes in CKD and kidney transplantation, a deeper understanding of the specific microbial alterations associated with renal replacement therapies is warranted. Few studies have directly compared the gut microbial composition across hemodialysis and kidney transplant populations, and even fewer have included healthy individuals as controls. Moreover, considering that diet is a key modulator of gut microbiota, we included dietary questionnaires to account for potential confounding factors related to nutrient intake.

In this study, we aimed to characterize and compare the gut microbiota profiles of patients undergoing hemodialysis, kidney transplant recipients, and healthy controls. We explored differences in microbial diversity, taxonomic composition, and potential associations with clinical and dietary parameters.

## 2. Materials and Methods

### 2.1. Study Design and Participants

This study was designed as a prospective observational cohort study. Participants were recruited from the Nephrology and Renal Transplant outpatient clinics of “Dr. C.I. Parhon” Clinical Hospital and from affiliated dialysis units in Iași, Romania.

Inclusion criteria were: Age > 18 years and a diagnosis of CKD stage 5D in a hemodialysis program or kidney transplantation for a minimum period of three months. The etiology of renal disease is presented in Supplementary Table S1.

Exclusion criteria included: Active malignancy, pregnancy, active systemic infection (due to potential confounding effects on inflammatory biomarkers), severe malnutrition, congenital heart disease, presence of pacemakers or metal implants, limb amputation, or antibiotic therapy, probiotics, biologics, or prebiotic supplements within 3 months before enrolment and stool collection. Participants were also screened for autoimmune disorders, inflammatory bowel disease, and celiac disease, and none of the included subjects had these conditions.

The TX group included adult recipients with stable graft function, at least 3 months post-transplantation, lacking recent rejection episodes or infectious complications—the mean time since transplantation was approximately 8 years. Exclusion criteria included acute infection or hospitalization in the previous 3 months, recent antibiotic/probiotic use (<3 months), active autoimmune or gastrointestinal inflammatory disease, and inability to provide a valid stool sample. All kidney transplant recipients received induction immunosuppression according to standard institutional and national protocols at the time of transplantation, most commonly with an interleukin-2 receptor antagonist (basiliximab) or antithymocyte globulin in high-risk cases. Maintenance therapy included a calcineurin inhibitor (tacrolimus or cyclosporine), an antimetabolite (mycophenolate mofetil or azathioprine), and corticosteroids—see Supplementary Table S2.

**Figure 1:**
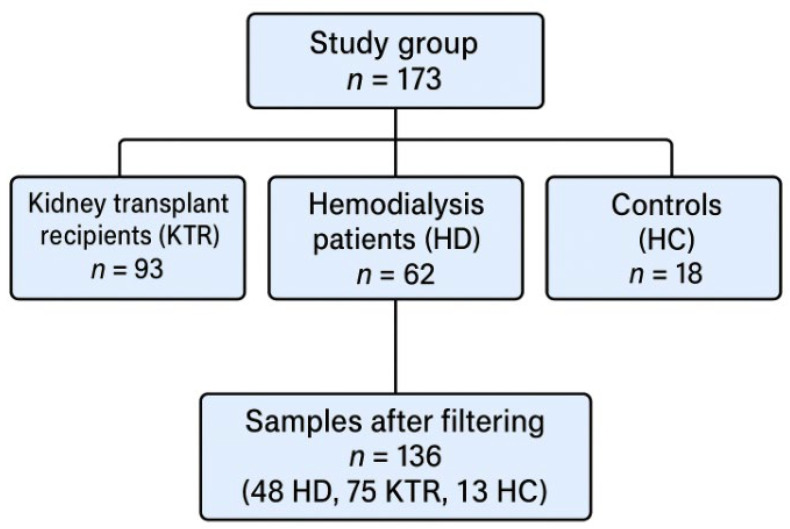
Flow diagram of participants in the study.

All potential participants underwent a preliminary discussion to assess eligibility, including recent infections and antibiotic use. Eligible individuals were invited to a baseline evaluation that included a detailed clinical interview conducted by a trained member of the research team. The study group comprised 173 eligible participants: 93 kidney transplant recipients (KTR), 62 hemodialysis patients (HD), and 18 controls (HC) (see Figure 1). This was an exploratory, observational study designed to compare the gut microbiome composition among patients on maintenance hemodialysis and kidney transplant recipients. No formal sample size calculation was performed. The number of patients included in each group was determined by the availability of eligible participants during the recruitment period and by the successful extraction of microbial DNA from stool samples. Stool samples were collected from all consenting participants, but in some cases, DNA extraction failed to yield material of sufficient quality or quantity for sequencing. Consequently, the final number of subjects differed slightly between study groups. Despite these variations, the resulting sample size provided adequate statistical power to detect significant differences in microbial diversity and taxonomic composition among the three populations.

The study protocol was approved by the Research Ethics Committee of the “Grigore T. Popa” University of Medicine and Pharmacy, Iași, Romania (approval no. 227/25 September 2022). The study was conducted in accordance with the Declaration of Helsinki, national research ethics legislation (Law no. 206/2004), the National Ethics Council guidelines on scientific integrity (2020), and the European Union’s data protection regulations (GDPR, 2016/679). All participants provided written informed consent before enrolment.

### 2.2. Baseline Data

Demographic, clinical, biological, dietary, and treatment-related data were collected from hospital and dialysis units for all CKD patients. The etiologies of CKD are detailed for all study groups. A complete distribution across the hemodialysis and kidney transplant subgroups is provided in Supplementary Table S1. Treatment-related data are also provided in Supplementary Table S2. The most frequent therapies included ACE inhibitors or ARBs, beta-blockers, calcium channel blockers, mineralocorticoid receptor antagonists, statins, and, in kidney transplant recipients, triple immunosuppressive therapy based on tacrolimus, mycophenolate mofetil, and corticosteroids. Data on erythropoiesis-stimulating agents and phosphate binders were not systematically collected.

For the dialysis group, dialysis vintage was recorded. For the transplant group, additional data included pre-transplant dialysis vintage, date of transplantation, donor type (living or deceased), and current chronic immunosuppressive regimen. Diabetes mellitus was defined by self-reported history or by the use of oral antidiabetic drugs or insulin. Among the KT patients in our study, 16 patients (21%) had diabetes mellitus. Of these, 14 had diabetic kidney disease as the primary cause of CKD, while two patients developed post-transplant diabetes mellitus through follow-up. All diabetic patients were clinically stable and under adequate metabolic control at the time of microbiota sampling.

Cardiovascular disease (CVD) was described as a history of coronary artery disease (documented by coronary angiography or myocardial infarction), New York Heart Association (NYHA) class III/IV heart failure, or stroke.

Fasting blood samples were collected and stored at −80 °C until analysis. Inflammatory status, including C-reactive protein (CRP), was assessed using routine laboratory investigations.

Dietary intake was assessed using a structured Food Frequency Questionnaire (FFQ) adapted from the KDOQI Clinical Practice Guideline for Nutrition in CKD [15]. The questionnaire covered 12 components—fruits, vegetables, legumes, whole grains, red/processed meat, fish, dairy, sweets, fast food, sugary drinks, alcohol, and smoking exposure—with responses recorded on ordinal frequency scales (never/rarely, monthly, weekly, daily). The responses were numerically encoded and used to compute a Manhattan distance matrix. Differences among groups were analyzed via PERMANOVA (999 permutations), while associations between dietary patterns and microbial composition were tested using Mantel correlation (Spearman, 999 permutations).

### 2.3. Fecal Microbiota DNA Extraction, Amplification, and Sequencing

Human fecal material was sampled in 2 mL sterile centrifuge tubes and frozen immediately upon collection at −20 °C. The stool samples were subsequently transported on dry ice to the molecular biology laboratory, where they were kept at −80 °C until further processing.

### 2.4. DNA Isolation

To minimize the risk of cross-contamination, DNA extraction was carefully optimized and conducted in batches of 16 samples. Approximately 300 mg of fecal material was used for DNA isolation, employing the NucleoSpin^®^ Soil kit (Macherey-Nagel, Düren, Germany) with additional refinement steps. To enhance the breakdown of both Gram-positive and Gram-negative bacteria, the lysis protocol was optimized by incubating samples for 20 h at 37 °C with 800 µL of SL2 and 40 µL of proteinase K (20 mg/mL~600 U/mL) (Thermo Fisher Scientific, Waltham, MA, USA). This was followed by a 1-h incubation at 37 °C with 50 µL of lysozyme (10 mg/mL ~ 40 KU/mL) and 3 µL of lysostaphin (1 mg/mL~3 KU/mL) (Sigma-Aldrich Co., St. Louis, MO, USA). Samples were vigorously vortexed after each reagent was added. Subsequent mechanical lysis was performed using a FastPrep-24 homogenizer (MP Biomedicals, Irvine, CA, USA) at 6 m/s for 80 s, with ceramic beads from MN Bead Tubes type A from the NucleoSpin Soil kit. The lysed fecal material was centrifuged twice at 11,000× *g* for two minutes, according to the manufacturer’s protocol, to produce a transparent supernatant. Approximately 800 µL of supernatant was transferred to a NucleoSpin^®^ inhibitor removal column for binding, washing, and elution. Once the SE elution buffer was heated to 80 °C, 30 µL of genomic DNA (gDNA) was eluted, measured, and stored frozen at −20 °C until needed. An in-house mock community served as a positive control to assess the accuracy of the DNA extraction method, as previously described [16].

### 2.5. Quantification and Assessment of DNA Purity

The yield and purity of the DNA samples were determined by a Nanodrop spectrophotometer (Thermo Fisher Scientific, Waltham, MA, USA). The integrity and size of the DNA samples were assessed by nucleic acid electrophoresis. Agarose gels were performed using a 2% (*w*/*v*) solution of agarose stained with ethidium bromide in buffer TAE (40 mmol/L Tris-acetate, 1 mmol/L EDTA). Electrophoresis was performed at 180 mV for 30 min to separate samples by molecular weight.

### 2.6. 16S V3–V4 rRNA Gene Sequencing

The microbial 16S rRNA profiles of the fecal DNA extracts were analyzed using MiSeq 16S rRNA gene sequencing methods (Illumina, San Diego, CA, USA) that targeted the V3–V4 hypervariable regions of the 16S rRNA gene [17]. In brief, the extracted DNA samples were used to amplify the V3–V4 region of the 16S rRNA gene with specific primers with overhang adapters F: 5′-TCGTCGGCAGCGTCAGATGTGTATAAGAGACAGCCTACGGGNGGCWGCAG-3′ and R: 5′-GTCTCGTGGGCTCGGAGATGTGTATAAGAGACAGGACTACHVGGGTATCTAATCC-3′ that yielded an amplicon of approximately ~460 bp [18]. The amplification mix consisted of 12.5 µL 2× KAPA HiFi HotStart Ready Mix (Roche Holding AG, Basel, Switzerland), 2.5 µL of microbial genomic DNA and 5 µL of each forward and reverse 1 mM primer After denaturation (95 °C; 30 min), 25 cycles were performed consisting of denaturation (95 °C; 30 s), annealing (55 °C; 30 s), extension (72 °C; 30 s), and a final hold (72 °C; 5 min). Purification was performed using Agencourt AMPure XP magnetic beads on a BIOMEK FXP automatic workstation (Beckman Coulter, Brea, CA, USA). A second PCR was performed using dual indexes and adapters from the Nextera XT Index Kit V2 set A (Illumina, San Diego, CA, USA), followed by eight cycles of thermal cycling. DNase-free water was used as a negative control during library preparation. The final library was cleaned with AMPure XP magnetic beads and quantified with a Qubit fluorometer using a 1× dsDNA High Sensitivity Assay Kit (Thermo Fisher Scientific, Waltham, MA, USA). Barcoded amplicon libraries were pooled equimolar at 4 nM, and paired-end sequencing was conducted using a V3 MiSeq reagent kit (600 cycles) on an Illumina MiSeq instrument (Illumina, San Diego, CA, USA).

### 2.7. Bioinformatics and Statistical Analysis

Sequence processing was conducted using the DADA2 pipeline [19] in R (version 4.3.2), with minor adjustments similar to those previously described [20,21]. After performing chimera detection and removal, taxonomy was assigned with an 80% bootstrap confidence cut-off using the RDP Naive Bayesian classifier implemented in dada2, against the SILVA database (release 138) [22]. Amplicon sequence variants (ASVs) unclassified at the phylum level, as well as those classified as eukaryotic, archaeal, mitochondrial, or chloroplast, were discarded. Samples with fewer than 5000 reads after taxonomical filtering were removed from further analyses (22.6% HD, 19.4% KTR, 27.8% HC). A total of 136 samples—48 from the HD group, 75 from the KTR group, and 13 from the control group —remained in the study after all filtering steps.

All statistical analyses were performed in R, and graphical representations were generated using ggplot2 v 3.5.1 [23]. Bacterial relative abundances were analyzed at the genus level, with differences in taxon abundances between groups being assessed using the Wilcoxon rank-sum test with false discovery rate (FDR) correction. Community composition barplots were created using the fantaxtic package (v 0.2.1). Relative abundances were also summarized in Supplementary Table S3 at phylum, family, and genus levels (median [IQR] %, prevalence ≥5%, abundance threshold 0.1%), with group differences tested by Kruskal–Wallis with Dunn’s post hoc (FDR). Core genera were defined as present in ≥80% of samples at ≥0.1% relative abundance within each patient group; the global core was defined as the intersection of the three group-specific cores. α-diversity metrics, including Chao1, Shannon, and Inverse Simpson indices, were calculated using the vegan package v 2.6-4 [24], while Faith’s phylogenetic diversity (Faith’s PD) was computed using the picante package v 1.8.2 [25]. Group comparisons for α-diversity were evaluated using Wilcoxon rank-sum tests with FDR correction. Post hoc power calculations for key effect sizes were performed using the pwr package v1.3-0.

Principal Component Analysis (PCA) and Sparse Partial Least Squares Linear Discriminant Analysis (sPLS-DA) were applied to centered log-ratio (CLR)-transformed genus-level data using the mixOmics package v 6.26.0 [26]. Linear Discriminant Analysis Effect Size (LEfSe) was performed using the microbiome Marker package v 1.8.0, with an LDA threshold of 3.7 and an adjusted Kruskal–Wallis *p*-value cutoff of 0.01.

To assess differences in dietary intake patterns among the three clinical groups, a dietary distance matrix was constructed from food frequency questionnaire responses. The questionnaire covered 12 components (fruit, vegetables, legumes, whole grains, red/processed meat, fish, dairy, sweets, fast food, sugary drinks, alcohol, smoking exposure). Responses were recorded on ordinal frequency scales (e.g., never/rarely, monthly, weekly, daily) and numerically encoded to calculate pairwise Manhattan distances, a procedure that has been previously used for diet frequency data [27]. The resulting distance matrix was analyzed using PERMANOVA (999 permutations), with clinical group as the independent variable. The association between dietary intake patterns and overall gut bacterial composition was tested via the vegan package using the Mantel test (Spearman correlation, 999 permutations), comparing the dietary distance matrix and the Aitchison distance matrix derived from CLR-transformed genus-level abundances. To explore potential relationships between diet and bacterial α-diversity, we first performed principal coordinate analysis (PCoA) using classical multidimensional scaling on the dietary distance matrix. The first principal coordinate (PC1) was then used to represent the dominant axis of dietary variation. Spearman’s rank correlation tests were then used to assess associations between PC1 and microbial α-diversity metrics (e.g., Inverse Simpson, Shannon, Faith’s PD, Chao1 indices).

## 3. Results

### 3.1. Anthropometric Parameters

Laboratory and clinical data revealed several differences between the HD and KTR groups (Table 1). Since no data were available for the controls other than sex and age, this group was excluded from comparisons of laboratory and clinical data. No significant sex differences were observed between the three study groups (χ^2^ = 1.49, *p* = 0.473). However, HD patients were on average older than KTR (mean age: 58.9 vs. 44.8 years, *p* < 0.001), and HC were the youngest (mean age: 27.2 ± 1.69, *p* < 0.001). Although the mean age differed significantly between groups (HD older than KTR, and HC the youngest), this difference was not considered relevant for the microbiota analysis, and the control group was excluded from clinical and laboratory comparisons, as they were young individuals without comorbidities. In addition, HD patients were more likely to suffer from congestive heart failure than KTR (mean prevalence: 54.17% vs. 10.67%, *p* < 0.001), as well as ischemic cardiomyopathy (mean prevalence: 45.83% vs. 8%, *p* < 0.001), and were more likely to have atrial fibrillation (mean prevalence: 27.08% vs. 2.66%, *p* < 0.001). No significant group differences were detected for serum albumin levels (*p* = 0.197). However, all other clinical parameters were different between the two CKD groups (Table 1).

### 3.2. Bacterial Composition

**Figure 2:**
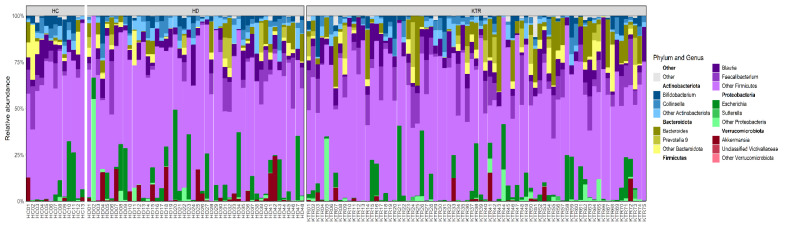
Comparative overview of dominant phylum and genus-level microbial composition in HC, HD and KTR individuals; corresponding phyla highlighted in bold above each genus group.

Successive sequence quality filtering stages reduced the number of participants from 173 to 136, resulting in a study group composed of 48 patients with Hemodialysis (HD, ♂31:17♀), 75 kidney transplant recipients (KTR♂44:31♀), and 13 controls (HC, ♂6:7♀) (see Figure 1). In total, 6,212,905 sequences were read across all 173 samples (average: 35,912, median: 20,688 reads/sample), with 56.92% of the sequences remaining after consecutive filtering steps. The final table contained 493 unique bacterial sequences for all 136 samples. Figure 2 shows the abundance of the two most abundant bacterial genera from the five most abundant phyla in each analyzed sample. Supplementary Table S3 presents the relative abundances of the main bacterial taxa at the phylum, family, and genus levels, together with the results of statistical comparisons between study groups (HD, KTR, and HC).

In general, the phyla Firmicutes and Bacteroidota dominate across all sample types, with varying proportions of Actinobacteriota, Proteobacteria, and Verrucomicrobiota, similar to what has been previously reported in CKD patients [28]. HD samples showed a slightly higher prevalence of Proteobacteria than HC and KTR samples. Still, there were no significant differences in the relative abundance of genera within this phylum between groups. The genus *Akkermansia,* from the Verrucomicrobiota phylum, showed variable presence across samples, being significantly more abundant in HD than in KTR samples (3.17 ± 0.87% in HD vs. 0.91 ± 0.33% in KTR; FDR-adjusted *p* = 0.022). Overall, the phylum Verrucomicrobiota was significantly different between the HD and KTR samples (*p* < 0.001). No other significant differences were found at the phylum level. A comprehensive genus-level summary, annotated with higher taxonomic ranks and including per-group medians, prevalence, and statistical tests, is provided in Supplementary Table S3.

Using a prevalence threshold of ≥80% at ≥0.1% relative abundance, the global core of microbiota genera (shared by HC, HD, and KTR) comprised *Blautia*, *Dorea*, *Subdoligranulum*, the *Eubacterium hallii* group, and Unclassified Lachnospiraceae. Group-specific cores differed modestly: HC additionally included *Bifidobacterium*, *Coprococcus*, *Faecalibacterium*, *Streptococcus*, *Escherichia*, *Fusicatenibacter*, Erysipelotrichaceae UCG-003 and *Eubacterium coprostanoligenes* group; HD additionally included *Anaerostipes*, *Ruminococcus torques* group and *Eubacterium coprostanoligenes* group; KTR additionally included *Escherichia*, *Faecalibacterium*, *Fusicatenibacter*, *Streptococcus*, and *Ruminococcus torques* group. These findings indicate a broadly shared backbone with limited group-specific additions.

**Figure 3:**
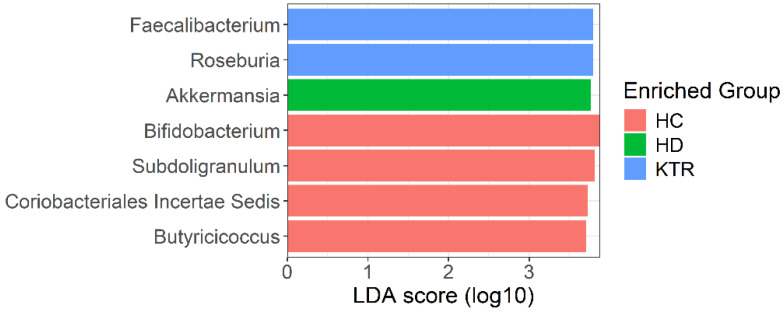
Results of differential abundance analysis by LEfSe after normalizing abundances by VST transformation at the genus level; LDA threshold = 3.7; adjusted *p* < 0.01, Kruskal–Wallis tests with BH correction.

Differential abundance analysis using the LEfSe method identified seven genera with LDA scores > 3.7 (adjusted *p*-values < 0.01), indicating significant differences between groups (Figure 3). Bacteria from the genera *Faecalibacterium* and *Roseburia* were more frequently detected in KTR samples than in HC and HD samples. In contrast, the genus *Akkermansia* was differentially abundant in HD samples. At the same time, bacteria from the genera *Bifidobacterium*, *Subdoligranulum*, *Butyricicoccus*, and *Coriobacteriales Incertae Sedis* were predominantly found in control patients (Figure 3).

**Figure 4:**
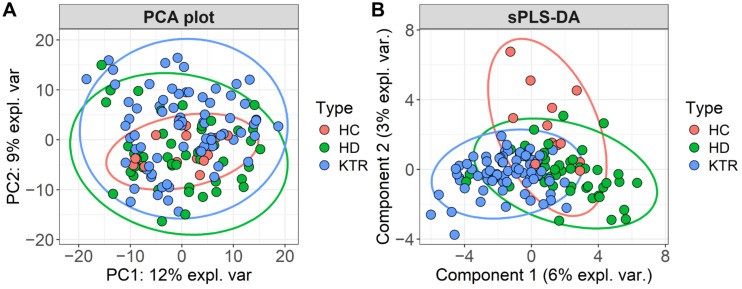
(**A**) PCA analysis and (**B**) sPLS-DA bi-plot from CLR-transformed genus abundance data.

Principal Component Analysis (PCA) on CLR-transformed abundance data did not reveal group separation based on bacterial composition, and a notable overlap was observed among all three groups (Figure 4A). Thus, the primary source of variation could clearly not be attributable to sample type. We therefore applied the sPLS-DA model to identify more effective microbial features that differentiate the three study groups, achieving better separation (Figure 4B). However, only a small proportion of the variation could be explained by the model components.

**Figure 5:**
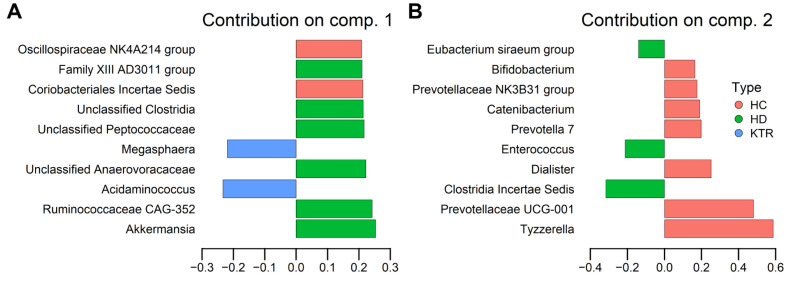
sPLS-DA loadings on genus-level CLR-transformed data. (**A**) Component 1. (**B**) Component 2. Top 10 contributing taxa per component are shown. Bars show how strongly each genus contributes to the component. *X*-axis represents loading weight. Signs of loadings correspond to the direction of group separation in the sPLS-DA bi-plot. Bars are colored according to group.

While there is some overlap between the clusters, each group has a distinct centroid and spread. Notably, HC samples exhibit greater separation from HD and KTR, with several notable differences in component contributions (Figure 5).

LEfSe highlighted taxa with firm univariate shifts between groups (e.g., *Akkermansia* enriched in HD; *Faecalibacterium* and *Roseburia* in KTR), whereas sPLS-DA emphasized taxa that jointly optimize group discrimination in a compositional multivariate space (e.g., contributions from *Akkermansia* and genera from the class Clostridia in HD and *Acidaminococcus*/*Megasphaera* in KTR and *Tyzzerela* in HC). Consequently, the two approaches can emphasize partially overlapping but non-identical taxa. As such, the differences reflect method design rather than inconsistency.

### 3.3. Bacterial Diversity

**Figure 6:**
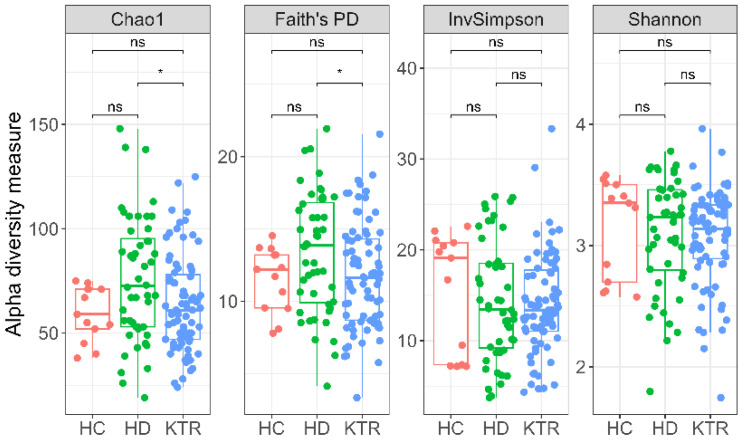
Alpha diversity metrics: Chao1 index, Faith’s phylogenetic diversity, inverse Simpson index, and Shannon index; * *p* < 0.05; ns—not significant (Wilcoxon rank-sum test).

We found no significant differences between the HC group and the two patient study groups in phylogenetic diversity, as measured by Faith’s PD, or in other calculated α-diversity indices (all FDR-adjusted *p* > 0.05). Given the small HC sample size (*n* = 13), a post hoc sensitivity analysis indicated ~60% power to detect a large HC vs. HD effect on the Chao1 index (d = 0.71) at α = 0.05. Thus, the non-significant result HC vs. HD is underpowered/inconclusive rather than evidence of no difference. In contrast, the HD group was more phylogenetically diverse than the KTR group (FRD-adjusted *p =* 0.025, Figure 6). Additionally, species richness, as reflected by the Chao1 index, was significantly higher in HD patients compared to KTR (FDR-adjusted *p* = 0.029).

### 3.4. Impact of Diet

**Figure 7:**
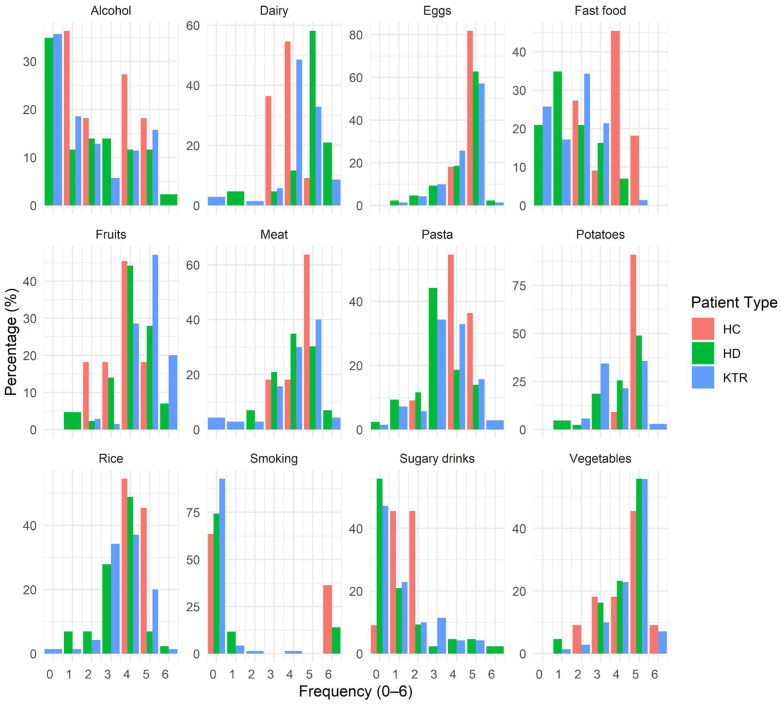
Dietary frequency distributions by group. Each panel shows one dietary item from the food frequency questionnaire; the *X*-axis is the ordinal frequency score 0–6 (0—never; 1—rarely; 2—once a month; 3—2–3 times a month; 4—once a week; 5—3–4 times a week; 6—daily) and the *Y*-axis is the percentage of respondents in that group reporting that score. Bars are colored by patient group. Within each panel, percentages for a given group sum to ~100% across the 0–6 categories (absent categories plot at 0%). Sample sizes: HC *n* = 13, HD *n* = 48, KTR *n* = 75.

Correlation analysis between the first principal coordinate of dietary variation and microbial α-diversity indices showed no significant results (e.g., Inverse Simpson vs. diet PC1: ρ = −0.147, *p* = 0.104), suggesting that overall dietary profiles do not explain variations in bacterial diversity. As such, diet, as assessed through our dietary questionnaire, does not substantially influence microbiome composition or diversity. However, PERMANOVA analysis revealed that the clinical group was a significant determinant of dietary intake patterns (R^2^ = 0.0906, *p* = 0.001), with over 9% of the variance in diet composition explained by clinical status (HD, KTR, or HC). Dietary patterns followed expected clinical trends: the HC group reported a dietary pattern consistent with Western-type nutritional habits, characterized by higher intake of meat, processed foods, sugary drinks, and alcohol, as well as greater smoking frequency (Figure 7); in contrast, the KTR group reported on average more fruit and vegetable intake, while HD patients had the highest intake of dairy products and reported the lowest frequency of fast-food consumption. These trends align with known dietary recommendations and lifestyle constraints for CKD and post-transplant management.

## 4. Discussion

This study presents a comprehensive analysis of gut microbiota in patients undergoing hemodialysis and kidney transplant recipients. Both patient groups exhibited changes in gut microbial composition compared with healthy controls, with distinct microbial signatures between HD and KTR. Specifically, HD patients demonstrated a reduction in SCFA-producing taxa and an enrichment of bacterial families linked to dysbiosis and inflammation (*Peptococcaceae*, *Anaerovoracaceae*), as well as *Akkermansia*, a bacterial genus primarily linked to acetate/propionate production. On the other hand, KTR patients displayed partial recovery of SCFA producers. However, their microbiota remained distinct from that of healthy individuals.

### 4.1. Key Taxa Driving Group Separation

In the HD group, the genera *Akkermansia*, CAG-352 (Ruminococcaceae), Family XIII AD3011 group (Anaerovoracaceae), unclassified Anaerovoracaceae and Peptococcaceae, and other unclassified bacteria of the Clostridia class were the principal contributors to separation along component 1. Notably, CAG-352 has been associated with intestinal polyps and mucosal damage [29]. At the same time, the prevalence of Anaerovoracaceae has been linked to diabetic retinopathy, reflecting shared pathogenic mechanisms between kidney disease and diabetic complications [30]. Specific Peptococcaceae genera are more abundant in non-alcoholic fatty liver disease, a known risk factor for incident CKD [31].

Interestingly, bacteria from the genus *Akkermansia*, which are generally considered a marker of gut health due to their metabolic, protective, and immune-regulating functions [32], were consistently found in higher levels in HD patients. Since disruption of intestinal mucosa integrity by an exaggerated immune response to gut microbiota contributes to the development of chronic idiopathic inflammatory diseases, conditions which are often alleviated in subjects with higher gut levels of *Akkermansia*, this finding could suggest that the gut microbiome profile of HD patients is more favorable compared to KTR in terms of overall gut health. However, it has been recently pointed out that specific antidiabetic medication can increase levels of *Akkermansia*, potentially suggesting a medication-induced restoration of gut dysbiosis associated with type 2 diabetes [33]. In our cohort, however, we found no significant difference in *Akkermansia* abundance between HD patients with and without diabetes, even after adjusting for sex and age as confounders (*p* = 0.71). This suggests that the *Akkermansia* enrichment observed in HD patients is unlikely to be driven by diabetes status or its pharmacological management, but more likely a CKD/HD-related physiological effect. In addition, the abundance of *Akkermansia* did not correlate with any laboratory parameters in HD patients (*p* > 0.05), nor with the first dietary ordination axis (ρ = 0.25, *p* = 0.113).

In contrast, the genera *Acidaminococcus* and *Megasphaera* were associated with KTR group separation on component 1. Both genera have previously been reported as more prevalent in patients with diabetic nephropathy [34], diabetic retinopathy and obesity [35]. Additionally, elevated levels of bacteria belonging to the genus *Megasphaera* have previously been associated with insulin resistance in patients with diabetic neuropathy [36]. While we did not assess insulin resistance in our KTR group, nor complications in diabetic KTR specifically, this could serve as an interesting direction for future research. Importantly, the prevalence of diabetes did not differ significantly between HD and KTR patients in our cohort (χ^2^ = 0.853, *p* = 0.335), suggesting that the identified microbial differences are not solely explained by diabetes status. Instead, the transplant-related metabolic environment, which includes recovery from uremic conditions, exposure to immunosuppressive therapy, and less stringent dietary restrictions than in HD, may favor the growth of taxa such as *Acidaminococcus* and *Megasphaera*. These observations highlight the importance of future studies to identify the relative contributions of metabolic status, immunosuppressive medications, and diet to post-transplant gut microbiome profiles.

Drivers of HC group delimitation on component 2 included, most notably, bacteria from the genera *Tyzzerella* and Prevotellaceae UCG-001. The genus *Tyzzerella* has recently been identified as differentially abundant in patients with HC compared to those with CKD [37]. Still, an association between its increased abundance and poor dietary quality has been observed in healthy adults [38]. Similarly, higher relative abundances of Prevotellaceae UCG-001 have been previously associated with thin, healthy individuals and with a fiber- and carbohydrate-rich diet [39]. Thus, the enrichment of these genera in the HC group may reflect distinct dietary and lifestyle patterns compared with those in HD and KTR. However, formal testing in our study group showed that overall dietary profiles do not explain microbiome composition variation (Mantel test, r = −0.021, *p* = 0.626). These results suggest that, although specific taxa in HC are consistent with distinct diet- and lifestyle-linked signatures, diet does not structure microbiome composition in our cohort.

### 4.2. Microbiota Composition Patterns

Our results largely align with previous studies, which show that Firmicutes and Bacteroidota dominate the overall gut microbiota across all study groups. Verrucomicrobiota was the only differentially abundant phylum between groups, being increased in HD patients. We observed increased Proteobacteria in HD and higher abundances of *Faecalibacterium* and *Roseburia* in KTR. In contrast, HC were enriched in *Bifidobacterium*, *Tyzzerella*, and *Prevotellaceae UCG-001*, reflecting most likely different lifestyles.

The enrichment of SCFA producers in KTR may reflect partial reversal of uremia-induced dysbiosis, while immunosuppressive therapy and metabolic comorbidities introduce new selective pressures.

In HD patients, Clostridia-related taxa (Anaerovoracaceae, Peptococcaceae, unclassified Clostridia) were enriched, along with *Akkermansia*. Previous studies have linked *Clostridium innocuum*, *C. symbiosum*, *Hungatella hathewayi*, and *Ruminococcus gnavus* to the production of uremic toxins, barrier disruption, and increased mortality in CKD and transplant populations [40,41]. Our results, therefore, align with broader evidence that dialysis patients maintain a microbiome biased toward taxa with potentially deleterious functional repertoires. The unexpected enrichment of *Akkermansia* in HD is noteworthy, as it is typically reduced in CKD and metabolic diseases. Only one prior study reported elevated *Akkermansia* in CKD, associating it with serum creatinine and estimated glomerular filtration rate (eGFR), though we observed no correlation with biochemical parameters in our HD cohort [42].

KTR had higher abundances of *Faecalibacterium* and *Roseburia*, SCFA-producing genera linked to metabolic and cardiovascular health, as well as *Acidaminococcus* and *Megasphaera*, taxa associated with obesity, diabetic nephropathy, and insulin resistance [43]. While the clinical significance of these findings in transplant recipients remains uncertain, they may reflect the complex metabolic milieu shaped by immunosuppressive therapy, recovery from uremia, and more liberalized dietary intake. Future work should explore whether these genera contribute to post-transplant metabolic complications or represent transient shifts in the early post-transplant period.

Alpha-diversity analyses showed higher phylogenetic diversity (Faith’s PD) and species richness (Chao1) in HD compared with KTR, while correlations with serum biomarkers or diet were generally weak.

Taken together, our results are broadly consistent with the prior literature in showing that CKD and transplant populations share a dysbiotic signature characterized by the depletion of SCFA producers and the enrichment of taxa with pathogenic or UT-generating potential. The recovery of specific beneficial genera in KTR supports the notion that transplantation may partially reverse uremia-associated dysbiosis, although immunosuppression and metabolic comorbidities introduce new ecological pressures. The case of *Akkermansia* highlights the importance of contextual interpretation, as its role may vary depending on disease state, treatment exposure, and environmental niche. These findings underscore the importance of integrative approaches that combine taxonomic, functional, and clinical data to elucidate the multifaceted determinants of microbiome structure in advanced kidney disease.

Our findings have several implications for the management of patients with CKD and transplantation.

First, gut microbiota composition may serve as a biomarker of long-term outcomes. As shown in both population-based and transplant-specific cohorts, indices of dysbiosis—such as reduced diversity, dissimilarity to healthy controls, and enrichment of pathobionts—are consistently predictive of mortality [44]. Swarte et al. recently showed that, in a cohort of 1337 solid organ transplant recipients, greater distance of the microbiome from the general population, reduced Shannon diversity, and increased richness of antibiotic resistance genes and virulence factors were all independently associated with higher all-cause and cause-specific mortality [45]. Incorporating microbiome profiling into risk stratification tools may help identify patients at high risk who could benefit from closer monitoring or targeted interventions.

Second, therapeutic modulation of the microbiota holds promise. Dietary strategies to increase fiber intake, prebiotic supplementation, and the use of probiotics or next-generation commensals (e.g., *F. prausnitzii*, *Akkermansia muciniphila*) could theoretically restore SCFA production and barrier integrity. Early pilot trials suggest that resistant starch supplementation reduces circulating uremic toxins in CKD, though robust RCTs in KTR are lacking. Fecal microbiota transplantation (FMT), though still experimental in the transplant setting, has shown efficacy in recurrent *Clostridioides difficile* infection and could be explored for broader applications [46].

### 4.3. Limitations of the Study

Our study has several limitations that should be acknowledged when interpreting its results. The number of subjects in the HC group was relatively small and not age-matched to the CKD patients under study. Additionally, clinical metadata were unavailable for this group, limiting comparisons to dietary questionnaires and microbial composition and diversity. Additionally, the cross-sectional design does not permit inference on temporal changes or causality between kidney disease, transplantation, and microbiome alterations. Finally, dietary intake was assessed through self-reported questionnaires, which may be subject to recall bias and do not fully reflect nutritional intake.

## 5. Conclusions

Our study demonstrates that both hemodialysis (HD) and kidney transplantation (KTR) are associated with distinct alterations in gut bacterial composition, reflecting disease-specific physiological pressures and treatment-related effects. While transplantation was associated with partial recovery of SCFA-producing taxa, the gut microbiota of HD patients remained enriched in bacteria associated with uremic toxin production. These findings underscore the persistence of dysbiosis despite therapeutic interventions and suggest a potential contribution to systemic inflammation and long-term clinical outcomes.

Although our study is limited by sample size and the selection of an age-matched control group, the results highlight the value of integrating microbiome profiling into kidney disease research. Such approaches may facilitate patient risk stratification and inform therapeutic strategies aimed at restoring beneficial microbial taxa, mitigating systemic inflammation, and ultimately improving long-term outcomes in both HD and KTR populations.

## Figures and Tables

**Table 1:** Anthropometric and clinical data of the CKD participants.

		HC (N = 13)	HD (N = 48)	KTR (N = 75)	*p*-Value
Gender	M:F	6:7	17:31	31:44	0.473 ^a^
Age (years)	M (SD)	27.2 ± 1.69	58.9 (13.2)	44.8 (10.4)	<0.001 ^b^
	Range	23–35	33–82	24–67	
Months from intervention	M (SD)	Not applicable	97 (68.9)	104 (80.9)	0.971 ^c^
	Range		4–340	3–288	
BSA (m^2^)	M (SD)	1.80 (0.21)	1.82 (0.207)	1.86 (0.214)	0.429 ^b^
	Range	1.35–2.1	1.38–2.2	1.49–2.37	
Albumin (g/L)	M (SD)	43.6 (4.6)	41.6 (9.6)	43.3 (5.07)	0.197 ^c^
	Range	39–48	36.7–56	26–54	
CRP (mg/L)	Median (Q1–Q3)	2.8 (1–4.5)	6.28 (2.68–12)	3.1 (2–5)	<0.001 ^c^
	Range	1–5.5	0.6–73	0.2–38	
Hemoglobin (g/dL)	M (SD)	13.3 (1.93)	11.2 (1.28)	13 (1.98)	<0.001 ^c^
	Range	11.5–16	8.9–16	9–17.2	

Data are expressed as mean (SD). BSA—body surface area; CRP—C-reactive protein; HD—hemodialysis; KTR—kidney transplant recipients; HC—healthy controls. The *p*-values represent comparisons among the three groups; ^a^—Chi-squared test, ^b^—*t*-test, ^c^—Wilcoxon rank-sum test. “Months from intervention” represents time since kidney transplantation or initiation of hemodialysis (not applicable for controls).

## Data Availability

The data presented in this study are available on request from the corresponding author.

## Supplementary Materials

The following supporting information can be downloaded at: https://www.mdpi.com/article/10.3390/jcm14228032/s1. Table S1: CKD etiology across the study subgroups; Table S2: Medication across the study subgroups; Table S3a: Relative abundance by phylum (HC, HD, KTR); Table S3b: Relative abundance by family (HC, HD, KTR); Table S3c: Relative abundance by family (HC, HD, KTR).
