# Supplementary material for: Distinct Gut Microbiome Signatures in Hemodialysis and Kidney Transplant Populations
Source: J Clin Med. 2025 Nov 12;14(22):8032. doi: 10.3390/jcm14228032 (PMC12653591; doi:10.3390/jcm14228032)
Supplement: Supplementary file 1 [file jcm-14-08032-s001.zip › jcm-3934277-supplementary Table S1 and S2.pdf]

Supplementary Table S1. CKD etiology across the study subgroups

Group	Etiology	n	% within group
HD		48	100%
HD	Other/unknown	15	31%
HD	Vascular nephropathy	9	19%
HD	Diabetic nephropathy	12	25%
HD	Chronic glomerulonephritis	7	14%
HD	Chronic tubulonephritis	5	10%
TX		75	100%
TX	Other/unknown	21	28%
TX	Chronic glomerulonephritis	31	41%
TX	Diabetic nephropathy	14	19%
TX	Vascular nephropathy	4	5%
TX	Chronic tubulonephritis	5	7%

Supplementary Table S2. Medication across the study subgroups

**Medication class**	**N patients**	**Patients (%)**
Antimetabolites (e.g., mycophenolate)	75	54.0
Corticosteroids	73	52.5
Statins	40	28.8
Mineralocorticoid receptor antagonists	39	28.1
Calcium channel blockers (BCC)	37	26.6
Beta-blockers	35	25.2
Centrally acting antihypertensive agents	21	15.1
Urate-lowering therapy (allopurinol)	19	13.7
Nitrates	16	11.5
Angiotensin receptor blockers (ARBs / sartans)	14	10.1
Antiplatelet agents	13	9.4
ACE inhibitors (IECA)	12	8.6
Anticoagulants	5	3.6
ARNI	4	2.9
Antiarrhythmic agents	3	2.2
Loop diuretics (furosemide)	2	1.4
